# Piezo1 Activation Drives Enhanced Collagen Synthesis in Aged Animal Skin Induced by Poly L-Lactic Acid Fillers

**DOI:** 10.3390/ijms25137232

**Published:** 2024-06-30

**Authors:** Kyung-A Byun, Je Hyuk Lee, So Young Lee, Seyeon Oh, Sosorburam Batsukh, Gwahn-woo Cheon, Dongun Lee, Jeong Hee Hong, Kuk Hui Son, Kyunghee Byun

**Affiliations:** 1 Department of Anatomy & Cell Biology, College of Medicine, Gachon University, Incheon 21936, Republic of Korea; 2 LIBON Inc., Incheon 22006, Republic of Korea; 3 Functional Cellular Networks Laboratory, Lee Gil Ya Cancer and Diabetes Institute, Gachon University, Incheon 21999, Republic of Korea; 4 Doctorbom Clinic, Seoul 06614, Republic of Korea; 5 Department of Thoracic and Cardiovascular Surgery, Gachon University Gil Medical Center, Gachon University, Incheon 21565, Republic of Korea; 6 Maylin Clinic, Pangyo 13529, Republic of Korea; 7 Department of Health Sciences and Technology, Gachon Advanced Institute for Health & Sciences and Technology (GAIHST), Gachon University, Incheon 21999, Republic of Koreaminicleo@gachon.ac.kr (J.H.H.)

**Keywords:** aged skin, collagen synthesis, intracellular Ca^2+^, Piezo1, poly L-lactic acid, senescence

## Abstract

Poly L-lactic acid (PLLA) fillers stimulate collagen synthesis by activating various immune cells and fibroblasts. Piezo1, an ion channel, responds to mechanical stimuli, including changes in extracellular matrix stiffness, by mediating Ca^2+^ influx. Given that elevated intracellular Ca^2+^ levels trigger signaling pathways associated with fibroblast proliferation, Piezo1 is a pivotal regulator of collagen synthesis and tissue fibrosis. The aim of the present study was to investigate the impact of PLLA on dermal collagen synthesis by activating Piezo1 in both an H_2_O_2_-induced cellular senescence model in vitro and aged animal skin in vivo. PLLA elevated intracellular Ca^2+^ levels in senescent fibroblasts, which was attenuated by the Piezo1 inhibitor GsMTx4. Furthermore, PLLA treatment increased the expression of phosphorylated ERK1/2 to total ERK1/2 (pERK1/2/ERK1/2) and phosphorylated AKT to total AKT (pAKT/AKT), indicating enhanced pathway activation. This was accompanied by upregulation of cell cycle-regulating proteins (CDK4 and cyclin D1), promoting the proliferation of senescent fibroblasts. Additionally, PLLA promoted the expression of phosphorylated mTOR/S6K1/4EBP1, TGF-β, and Collagen I/III in senescent fibroblasts, with GsMTx4 treatment mitigating these effects. In aged skin, PLLA treatment similarly upregulated the expression of pERK1/2/ERK1/2, pAKT/AKT, CDK4, cyclin D1, mTOR/S6K1/4EBP1, TGF-β, and Collagen I/III. In summary, our findings suggest Piezo1′s involvement in PLLA-induced collagen synthesis, mediated by heightened activation of cell proliferation signaling pathways such as pERK1/2/ERK1/2, pAKT/AKT, and phosphorylated mTOR/S6K1/4EBP1, underscoring the therapeutic potential of PLLA in tissue regeneration.

## 1. Introduction

Poly L-lactic acid (PLLA) filler has gained prominence in skin rejuvenation for its dual role in restoring facial volume, which tends to diminish with aging [1]. Moreover, PLLA fillers actively promote collagen synthesis as a bio-stimulant [2,3]. Collagen synthesis induced by PLLA fillers involves subclinical inflammation and a foreign body reaction initiated by various immune cells [3,4]. Upon injection into the skin, PLLA undergoes degradation into lactic acid [5], attracting various immune cells such as lymphocytes, macrophages, and giant cells to infiltrate around PLLA particles [5,6,7,8]. Furthermore, activated fibroblasts contribute to the production of extracellular matrix (ECM) materials, including collagen fibers, around PLLA particles [9].

In the context of skin rejuvenation, macrophages play diverse and crucial roles in the process of PLLA-induced collagen synthesis. They facilitate the infiltration of other immune cells and enhance the migration and proliferation of fibroblasts [10]. Additionally, macrophages secrete various cytokines—including transforming growth factor-beta (TGF-β)—which stimulate Collagen I and III synthesis [1]. Our previous research illustrated that PLLA fillers induce macrophage polarization toward the M2 phenotype, characterized by increased interleukin (IL)-10 and TGF-β levels, leading to the accumulation of collagen fibers in aged animal skin [2].

Piezo-type mechanosensitive ion channel component 1 (Piezo1) facilitates non-selective cation influx into the cytosol [11,12,13]. It is activated by stretch of cells’ membrane [14]. Piezo1 is activated either by positive or negative pressure over the cellular membrane [15]. This pressure may be originated by several stimuli such as compression and stiffness. It is also reported that Piezo1 is activated by tissue compression [16], changes in ECM stiffness [17], and shear flow [18].

Among cations, Piezo1 exhibits a preference for Ca^2+^ [19,20,21], potentially increasing cellular Ca^2+^ levels and activating various cell signaling pathways, such as those involved in cell proliferation [22]. Mechanical strain on macrophages activates Piezo1, leading to M2 polarization and the secretion of TGF-β. This results in increased proliferation and migration of bone marrow-derived mesenchymal stem cells, thereby enhancing osteogenesis [23].

In addition to TGF-β, Piezo1 activates various signaling pathways, including protein kinase B (AKT)/mechanistic target of rapamycin (mTOR), and p38 mitogen-activated protein kinases (MAPK), further contributing to cell proliferation [24,25,26].

In response to mechanical stretching, the AKT/mTOR signaling cascade is activated in osteoblasts and tendon cells [27,28]. This process leads to the phosphorylation of AKT and mTOR, subsequently increasing the phosphorylation of ribosomal protein S6 kinase-1 (S6K1) and eIF-4E-binding protein-1 (4EBP1) in tendon cells, ultimately resulting in increased collagen synthesis [29].

Moreover, mechanical stretching activates Piezo1, resulting in Ca^2+^ influx, the upregulation of ERK1, and the promotion of cell proliferation [14]. Elevated Ca^2+^ levels also drive cell cycle progression through the G1/S and G2/M transitions [29]. Piezo1 is involved in the cell cycle progression and proliferation of some cancer cells [30]. Inhibition of Piezo1 results in decreased levels of Cyclin D1 and Cyclin-Dependent Kinase 4 (CDK4), primary regulators of the G1/S transition [29,30].

While previous studies have demonstrated that mechanical stimuli induce collagen synthesis through the AKT/mTOR signaling pathway and Piezo1 increases cell proliferation in some tumors, the potential involvement of Piezo1 in PLLA-induced collagen synthesis remains unexplored. We hypothesized that mechanical stimuli may be altered when fibroblasts attach to PLLA particles, eventually activating Piezo1. Consequently, PLLA may induce Piezo1 activation, leading to the upregulation of ERK1/2, AKT, and mTOR, thereby enhancing fibroblast proliferation. Furthermore, we hypothesized that activated Piezo1 increases TGF-β, which promotes collagen synthesis. The aim of the present study was to investigate whether PLLA activated Piezo1, which increased collagen synthesis using in vitro models and aged animal skin.

## 2. Results

### 2.1. Poly L-lactic Acid Increases Intracellular Ca^2+^ Levels in Senescent Fibroblasts

**Figure 1:**
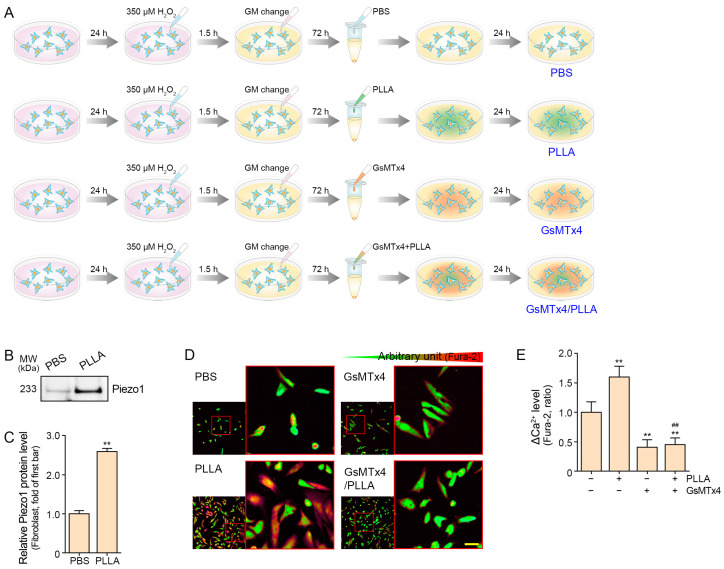
Regulation of intracellular Ca^2+^ ([Ca^2+^]_i_) and Piezo 1 by poly L-lactic acid (PLLA) in senescent CCD-986Sk fibroblasts. (**A**) Schematic diagram illustrating the treatment protocol for H_2_O_2_-induced senescent fibroblasts with PLLA and/or GsMTx4. (**B,C**) Western blot analysis of Piezo1 levels in senescent fibroblasts treated with PLLA. Quantitative assessment of Western blot data is presented as Piezo1. (**D**) Fluorescence images depicting Fura-2-stained senescent fibroblasts treated with PLLA and/or GsMTx4. The color gradient represents the intensity of Fura-2 staining. The red boxes are magnified image of the Fura-2-stained image. Scale bar = 20 µm (yellow dash). (**E**) Relative changes in [Ca^2+^]_i_ plateau are shown as the mean ± SD. **, *p* < 0.01 vs. first bar; ##, *p* < 0.01 second bar vs. fourth bar (Mann–Whitney U test). GsMTx4: Piezo1 inhibitor; PBS: phosphate-buffered saline; Piezo1: piezo-type mechanosensitive ion channel component 1.

H_2_O_2_-induced cellular senescence is a well-established in vitro model for studying aging mechanisms [31]. Senescence was induced in CCD-986Sk fibroblasts by treatment with 350 mM H_2_O_2_ for 1.5 h, and growth media were changed and incubated 72 h post-induction. The cells were collected and treated with PLLA, GsMTx4 (Piezo1 inhibitor), or phosphate-buffered saline (PBS) (as a control), and then incubated for 24 h before analysis (Figure 1A).

Expression of Piezo1 was increased by PLLA in the H_2_O_2_-treated fibroblasts (Figure 1B,C). To investigate the potential activation of Piezo1 by PLLA, we assessed cytosolic Ca^2+^ concentrations ([Ca^2+^]_i_) upon exposure to PLLA in senescent fibroblasts. The activity of Piezo1 and the effect of GsMTx4 were evaluated by Fura-2 fluorescence in CCD-986Sk fibroblasts [32,33] (Figure 1D). Our results revealed a significant increase in [Ca^2+^]_i_ levels in senescent fibroblasts treated with PLLA. Notably, in senescent fibroblasts simultaneously treated with GsMTx4, PLLA administration led to a reduction in [Ca^2+^]_i_ levels compared to the control group. Additionally, in GsMTx4-treated senescent fibroblasts, PLLA induced a slight increase in [Ca^2+^]_i_ levels, although this difference was not statistically significant (Figure 1E). These findings underscore the ability of PLLA to elevate [Ca^2+^]_i_ levels in senescent fibroblasts, implicating Piezo1 activation in mediating this effect and highlighting its role in the observed augmentation of [Ca^2+^]_i_ levels induced by PLLA.

### 2.2. Poly L-lactic Acid Enhances ERK1/2/AKT, CDK4, Cyclin D1, and Proliferation of Senescent Fibroblasts

Our investigation aimed to elucidate the role of Ca^2+^-linked cell signaling pathways in the proliferation of senescent fibroblasts.

**Figure 2:**
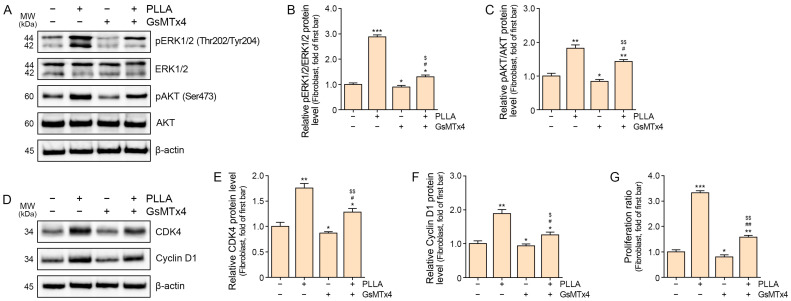
Regulation of ERK1/2/AKT, CDK4, and Cyclin D1 expression and proliferation by poly L-lactic acid (PLLA) in H_2_O_2_-induced senescent fibroblasts. (**A**–**C**) Western blot analysis of pERK1/2 (Thr202/Tyr204), ERK1/2, pAKT (Ser473), and AKT levels in senescent fibroblasts treated with PLLA and/or GsMTx4. Quantitative assessment of Western blot data is presented as pERK1/2/ERK1/2 (**B**), and pAKT/AKT ratios (**C**). (**D**–**F**) Western blot analysis of CDK4 and Cyclin D1 levels in senescent fibroblasts treated with PLLA and/or GsMTx4. Quantitative assessment of Western blot data is presented for CDK4 (**E**) and Cyclin D1 (**F**). (**G**) Cell proliferation in senescent fibroblasts following treatment with PLLA and/or GsMTx4 was measured using CCK-8 assays. Data are presented as the mean ± SD. *, *p* < 0.05; **, *p* < 0.01; ***, *p* < 0.001 vs. first bar; #, *p* < 0.05; ##, *p* < 0.01 second bar vs. fourth bar; $, *p* < 0.05; $$, *p* < 0.01 third bar vs. fourth bar (Mann–Whitney U test). AKT: protein kinase B; CDK4: cyclin-dependent kinase 4; ERK1/2: extracellular signal-regulated kinase 1/2; GsMTx4: Piezo1 inhibitor; MW: molecular weight; pAKT: phosphorylated AKT; pERK1/2: phosphorylated ERK1/2; Piezo1: piezo-type mechanosensitive ion channel component 1.

Treatment with PLLA resulted in a significant increase in the ratio of phosphorylated ERK1/2 to total ERK1/2 (pERK1/2/ERK1/2) and phosphorylated AKT to total AKT (pAKT/AKT) levels. Notably, administration of GsMTx4 attenuated the elevation of pERK1/2/ERK1/2 and pAKT/AKT ratios compared to control senescent fibroblasts. Although the PLLA-induced increase in these ratios was partially subdued in senescent fibroblasts treated with GsMTx4, the inhibition was less pronounced than in fibroblasts without GsMTx4 treatment (Figure 2A–C).

Furthermore, PLLA treatment led to upregulated CDK4 and Cyclin D1 expression in senescent fibroblasts. Conversely, GsMTx4 administration resulted in decreased levels of CDK4 and Cyclin D1 compared to control senescent fibroblasts. Similarly, in GsMTx4-treated senescent fibroblasts, the PLLA-induced increase in CDK4 and Cyclin D1 expression was mitigated, albeit to a lesser extent than in fibroblasts without GsMTx4 treatment (Figure 2D–F).

Additionally, PLLA treatment significantly enhanced the cell proliferation ratio in senescent fibroblasts. Conversely, GsMTx4 treatment reduced the cell proliferation ratio compared to control senescent fibroblasts. While GsMTx4 treatment attenuated the PLLA-induced increase in the cell proliferation ratio, the effect was less pronounced compared to senescent fibroblasts without GsMTx4 treatment (Figure 2G).

Overall, our findings highlight the pivotal role of PLLA-induced activation of Piezo1 and subsequent modulation of ERK/AKT signaling pathways in promoting the proliferation of senescent fibroblasts.

### 2.3. Poly L-lactic Acid Upregulates mTOR/S6K1/4EBP1, TGF-β, and COLLAGEN I/III Expression in Senescent Fibroblasts

**Figure 3:**
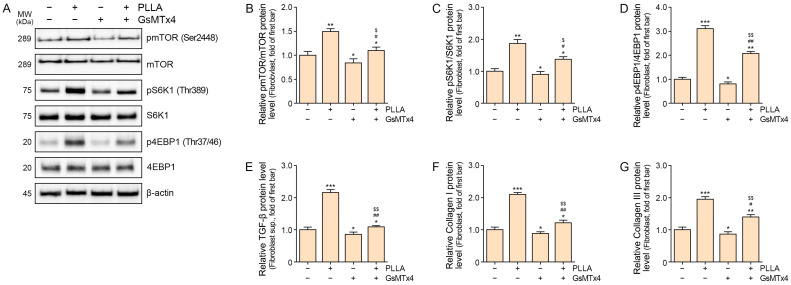
Regulation of mTOR/S6K1/4EBP1, TGF-β, and Collagen I/III expression by poly L-lactic acid (PLLA) in H_2_O_2_-induced senescent fibroblasts. (**A**–**D**) Western blot analysis of pmTOR (Ser2448), mTOR, pS6K1 (Thr389), S6K1, p4EBP1 (Thr37/46), and 4EBP1 levels in senescent fibroblasts treated with PLLA or GsMTx4. Quantitative assessment of Western blot data presented as pmTOR/mTOR (**B**), pS6K1/S6K1 (**C**), and p4EBP1/4EBP1 ratios (**D**). (**E**–**G**) Protein expression levels of secreted TGF-β, Collagen I, and Collagen III in senescent fibroblasts treated with PLLA or GsMTx4 were measured by ELISAs. Quantitative assessment of ELISA data presented for TGF-β (**E**), Collagen I (**F**), and Collagen III (**G**). Data are presented as the mean ± SD. *, *p* < 0.05; **, *p* < 0.01; ***, *p* < 0.001 vs. first bar; #, *p* < 0.05; ## *p* < 0.01 second bar vs. fourth bar; $, *p* < 0.05; $$, *p* < 0.01 third bar vs. fourth bar (Mann–Whitney U test). ELISA: enzyme-linked immunosorbent assay; GsMTx4: piezo1 inhibitor; mTOR: mechanistic target of rapamycin; MW: molecular weight; pmTOR: phosphorylated mTOR; PLLA: poly L-lactic acid; S6K1: ribosomal protein S6 kinase-1; 4EBP1: eIF-4E-binding protein-1.

To elucidate the effects of PLLA on senescent fibroblasts, we examined its impact on key proteins associated with mTOR signaling, including the pmTOR/mTOR, pS6K1/S6K1, and p4EBP1/4EBP1 ratios. GsMTx4 treatment decreased levels of these proteins compared to controls. However, PLLA administration in GsMTx4-treated senescent fibroblasts increased their expression, albeit less than in senescent fibroblasts without GsMTx4 treatment (Figure 3A–D).

Furthermore, PLLA influenced TGF-β and Collagen I/III expression in senescent fibroblasts. GsMTx4 treatment reduced TGF-β levels compared to controls, whereas PLLA increased TGF-β expression, though to a lesser extent in GsMTx4-treated senescent fibroblasts (Figure 3E). Similarly, Collagen I/III levels decreased with GsMTx4 treatment but increased with PLLA administration, though less prominently in GsMTx4-treated senescent fibroblasts (Figure 3F,G).

These findings illustrate PLLA’s capacity to modulate key proteins involved in mTOR signaling, TGF-β, and Collagen I/III expression in senescent fibroblasts, suggesting its potential role in regulating cellular processes associated with senescence and tissue remodeling.

### 2.4. Poly L-lactic Acid Increases ERK1/2/AKT, CDK4, and Cyclin D1 Expression and Proliferation of Fibroblasts in Aged Skin

**Figure 4:**
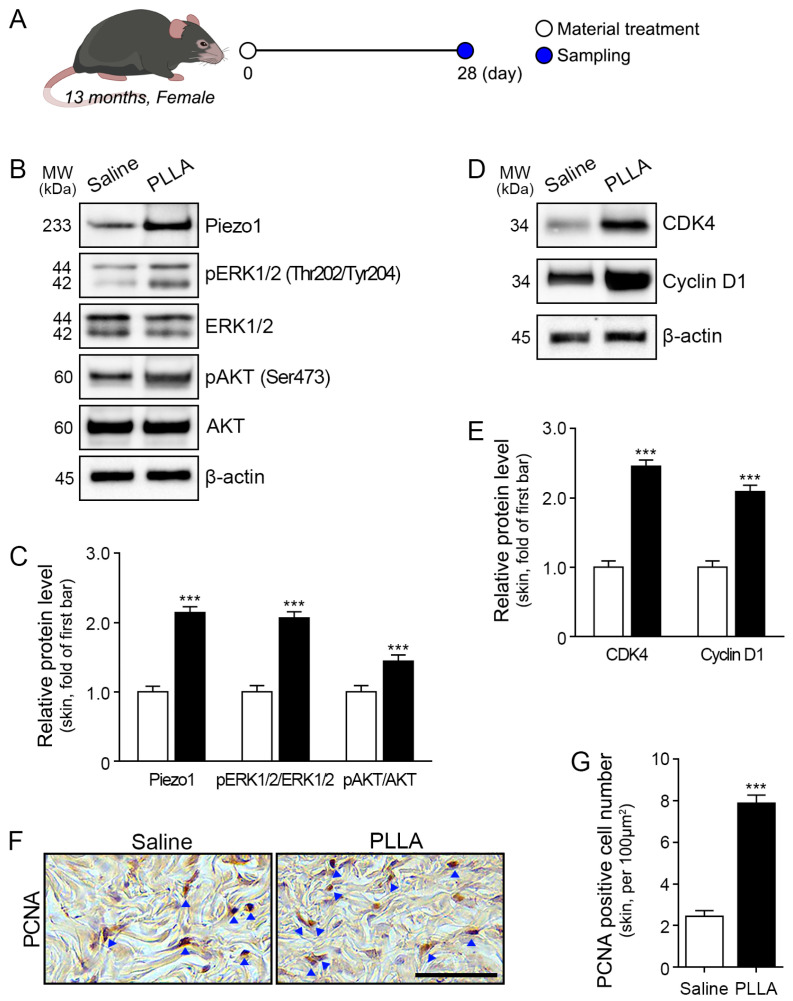
Regulation of Piezo1, ERK1/2/AKT, CDK4, and Cyclin D1 expression and proliferation of fibroblasts by poly L-lactic acid (PLLA) in aged skin. (**A**) Schematic diagram illustrating the treatment protocol for aged skin with saline or PLLA. (**B**) Western blot analysis of Piezo1, pERK1/2 (Thr 202/Tyr204), ERK1/2, pAKT (Ser473), and AKT levels in aged skin treated with PLLA. (**C**) Quantitative assessment of Western blot data presented as Piezo1, pERK1/2/ERK1/2, and pAKT/AKT ratios. (**D**) Western blot analysis of CDK4 and Cyclin D1 levels in aged skin treated with PLLA. (**E**) Quantitative assessment of Western blot data presented for CDK4 and Cyclin D1. (**F**) Fibroblast proliferation in aged skin treated with PLLA was measured by PCNA immunohistochemistry staining. Arrows indicate positive signals. Scale bar = 50 µm. (**G**) Quantitative assessment of immunohistochemistry data presented as the percentage of PCNA-positive fibroblasts. Data are presented as the mean ± standard deviation. ***, *p* < 0.001 vs. saline. AKT: protein kinase B; CDK4: cyclin-dependent kinase 4; ERK1/2: extracellular signal-regulated kinase; MW: molecular weight; pAKT: phosphorylated AKT; PCNA: proliferating cell nuclear antigen; pERK1/2: phosphorylated ERK1/2.

To further explore the effects of PLLA on aging skin, we investigated its impact on key signaling pathways and cellular processes (Figure 4A).

In aged animal skin, PLLA treatment increased Piezo1 (Figure 4B,C). In aged animal skin, PLLA treatment increased the pERK1/2/ERK1/2 and pAKT/AKT ratios (Figure 4B,C). Additionally, PLLA administration upregulated the expression of CDK4 and Cyclin D1 in aged skin (Figure 4D,E). Evaluation of the fibroblast proliferation ratio via proliferating cell nuclear antigen (PCNA) staining revealed an increase in PCNA-positive fibroblasts in PLLA-treated aged skin (Figure 4F,G).

These findings suggest that PLLA treatment induces a cascade of cellular events associated with enhanced proliferation and signaling in aged skin.

### 2.5. mTOR/S6K1/4EBP1, TGF-β, and Collagen I/III Expression Is Upregulated in Aged Skin

**Figure 5:**
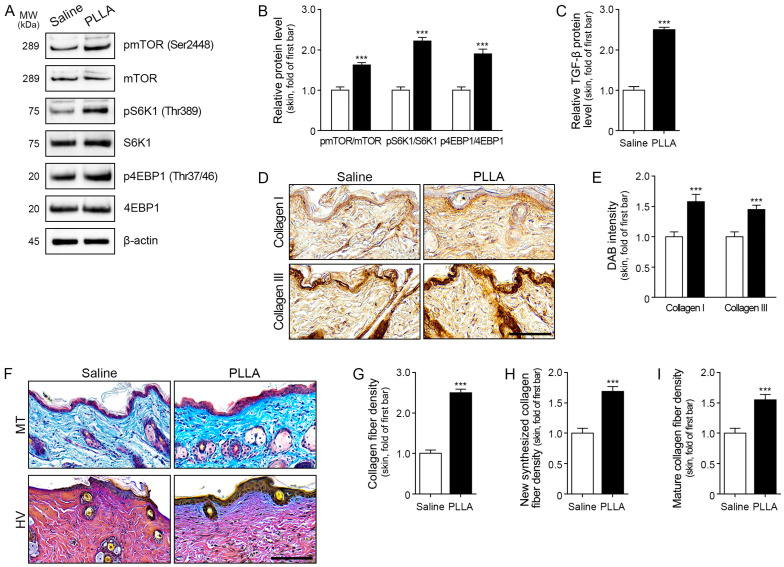
Regulation of mTOR/S6K1/4EBP1, TGF-β, and Collagen I/III expression by poly L-lactic acid (PLLA) in aged skin. (**A**) Western blot analysis of pmTOR (Ser2448), mTOR, pS6K1 (Thr389), S6K1, p4EBP1 (Thr37/46), and 4EBP1 levels in aged skin following PLLA treatment. (**B**) Quantitative assessment of Western blot data presented as pmTOR/mTOR, pS6K1/S6K1, and p4EBP1/4EBP1 ratios. (**C**) Protein expression levels of TGF-β in aged skin with PLLA treatment were measured by ELISA. (**D**) Protein expression levels of Collagen I and Collagen III in aged skin treated with PLLA were measured by immunohistochemical staining. Scale bar = 100 µm. (**E**) Quantitative assessment of immunohistochemical data for Collagen I and Collagen III. (**F**) Masson trichrome staining (upper) indicates total collagen fiber (blue), while Herovici staining (lower) distinguishes between newly synthesized collagen fibers (blue) and mature fibers (red). Scale bar = 100 µm. (**G**–**I**) Quantitative assessment of total collagen fiber (**G**), newly synthesized collagen fiber (**H**), and mature collagen fiber (**I**) densities. Data are presented as the mean ± SD. ***, *p* < 0.001 vs. saline. ELISA: enzyme-linked immunosorbent assay; HV: Herovici staining; mTOR: mechanistic target of rapamycin; MT: Masson trichrome staining; MW: molecular weight; pmTOR: phosphorylated mTOR; SD: standard deviation; S6K1: ribosomal protein S6 kinase-1; 4EBP1: eIF-4E-binding protein-1.

We conducted experiments to investigate the effects of PLLA on key signaling pathways and extracellular matrix components in aged skin. PLLA treatment led to an increase in the expression ratios of pmTOR/mTOR, pS6K1/S6K1, and p4EBP1/4EBP1 (Figure 5A,B). Additionally, TGF-β and Collagen I/III expression levels were elevated in aged skin following PLLA treatment (Figure 5C–E). Collagen fiber density, assessed by Masson trichrome staining, was also enhanced by PLLA treatment in aged skin (Figure 5F upper and G). Furthermore, Herovici staining was performed to differentiate between newly synthesized collagen (stained blue) and mature collagen (stained red) [34,35]. Both newly synthesized and mature collagen levels were elevated following PLLA treatment in aged skin (Figure 5F lower, H,I).

Overall, our findings demonstrate that PLLA treatment upregulates multiple signaling pathways and promotes collagen synthesis in aged skin, suggesting its potential as a therapeutic intervention for mitigating age-related changes and promoting skin rejuvenation.

## 3. Discussion

Piezo1 has been implicated in various fibrotic diseases, including pulmonary and renal fibrosis, through pathways involving TGF-β/SMAD or P38/MAPK [36,37]. However, its significance extends to skin conditions, notably in pathological skin fibrosis such as hypertrophic scars and keloid lesions of human tissue, where Piezo1 is overexpressed [38]. Additionally, in bleomycin-induced skin fibrosis lesions, Piezo1 expression is heightened, especially in α-SMA-positive myofibroblasts [38]. This activation of Piezo1 drives phenotypic changes in fibroblasts, leading to their differentiation into myofibroblasts and consequent enhancement in collagen synthesis, particularly types I and III [38]. Moreover, Piezo1 activation is responsive to changes in ECM stiffness [39]. In fibrosis, the excessive synthesis of ECM proteins by myofibroblasts increases ECM stiffness, further activating Piezo1 and perpetuating the cycle by promoting the synthesis of additional ECM proteins [38,39]. Given the pivotal role of Piezo1 in fibrotic processes, understanding its interaction with emerging skin rejuvenation therapies, such as PLLA fillers, is crucial for addressing skin aging and related concerns.

We hypothesized that fibroblasts might perceive the stiffness of PLLA differently compared to native dermal ECM, potentially triggering the activation of Piezo1, a mechanosensitive ion channel. Our results revealed expression of Piezo1 was increased by PLLA treatment in senescent fibroblasts. Activation of Piezo1 is associated with increased [Ca^2+^]_i_ levels in response to mechanical stimuli. To investigate this theory, we examined [Ca^2+^]_i_ levels in senescent fibroblasts after PLLA treatment. Our results revealed a significant increase in [Ca^2+^]_i_ levels upon PLLA exposure, indicating Piezo1 activation. Furthermore, administering GsMTx4, a Piezo1 inhibitor, resulted in decreased [Ca^2+^]_i_ levels, further implicating PLLA-induced activation in the observed elevation of [Ca^2+^]_i_. These findings provide initial evidence supporting our hypothesis that PLLA triggers Piezo1 activation, leading to heightened [Ca^2+^]_i_ levels within senescent fibroblasts.

To further elucidate the implications of PLLA-induced Piezo1 activation, we investigated its correlation with cell proliferation across various cell types. Piezo1 activation promotes cell proliferation in various cell types. For instance, increased Ca^2+^ influx through Piezo1 facilitates proliferation in pulmonary artery smooth muscle cells [40]. This effect is accompanied by an increase in PCNA-positive pulmonary artery smooth muscle cells and activation of the AKT/mTOR signaling pathway [40]. Inhibition of Piezo1, either through siRNA or GsMTx4 treatment, leads to decreased cell proliferation [40]. Similarly, Piezo1-induced Ca^2+^ influx has been implicated in the proliferation of muscle satellite cells [41]. Conversely, deletion of Piezo1 results in decreased myofiber regeneration following muscle injury [41]. Furthermore, macrophage proliferation is promoted through the upregulation of the Piezo1/AKT/Cyclin D pathway, while treatment with GsMTx4 leads to decreased macrophage proliferation [42]. The increase in [Ca^2+^]_i_ levels activates the ERK or AKT/mTOR pathways, which play crucial roles in cell proliferation and survival [43,44,45,46].

Building upon these findings, our study demonstrated that PLLA treatment increased phosphorylation of ERK and AKT in senescent fibroblasts, accompanied by elevated expression of CDK4 and Cyclin D1. Notably, administration of GsMTx4 mitigated these effects, indicating a pivotal role of Piezo1 in mediating PLLA-induced fibroblast proliferation through ERK and AKT activation.

Furthermore, mTOR, a key regulator of cell growth and metabolism, comprises two distinct complexes: mTORC1 and mTORC2 [47]. Phosphorylation of AKT activates mTORC1 [47,48], which, in turn, upregulates S6K1 and 4EBP1, known modulators of collagen synthesis [49,50]. The S6K1/4EBP1 pathway is intricately involved in increased Collagen I synthesis in mesenchymal stem cells and tendon cells [28,50], and inhibition of mTORC1 decreases collagen fiber synthesis [51]. Similarly, activation of the AKT pathway has been linked to increased collagen synthesis in fibroblasts and mesangial cells [52,53,54,55], while AKT inhibition leads to decreased collagen levels in dermal fibroblasts [52].

Our study results showed that PLLA induced phosphorylation of mTOR, S6K, and 4EBP1, which were decreased by administration of a Piezo1 inhibitor in senescent fibroblasts. Additionally, PLLA treatment increased Collagen I and III expression, which was diminished by the administration of GsMTx4 in senescent fibroblasts. These findings suggest that PLLA induces the synthesis of Collagen I and III, which might be promoted via Piezo1 activation. The decrease in phosphorylation of mTOR, S6K1, and 4EBP1 by the Piezo1 inhibitor occurred concurrently with PLLA treatment, indicating a potential regulatory role of Piezo1 in the mTOR signaling pathway.

TGF-β, a key regulator of skin aging processes, plays a significant role in upregulating the PI3K/AKT/mTOR pathway [56,57], leading to the phosphorylation of S6K and 4EBP1 [58]. TGF-β-induced activation of Piezo1 enhances collagen accumulation [20], a hallmark of aging skin. Interestingly, administration of a Piezo1 agonist (Yoda1) leads to increased TGF-β levels, contributing to fibrotic conditions such as renal fibrosis [20]. Additionally, mechanical stretch triggers Piezo1-induced Ca^2+^ influx in macrophages, leading to increased TGF-β secretion [23]. These findings underscore the intricate interplay between Piezo1, TGF-β signaling, and mechanical stimuli in modulating skin aging and fibrotic processes, suggesting potential therapeutic targets for skin rejuvenation and fibrotic diseases.

In our study, PLLA increased TGF-β secretion from senescent fibroblasts, a phenomenon that was mitigated by Piezo1 inhibition. TGF-β serves as a pivotal regulator of ECM protein synthesis, such as collagen, through various signal pathways. Specifically, TGF-β activates the canonical pathway involving SMAD2 and SMAD3, leading to increased expression of connective tissue growth factor and ECM protein synthesis [59]. Moreover, fibroblast proliferation is augmented through TGF-β-induced AKT activation [60,61]. Collagen synthesis and collagen fiber density play critical roles in skin aging and rejuvenation [62,63]. Reduced collagen production contributes to the formation of wrinkles, fine lines, and sagging skin [62,63]. In contrast, increased collagen synthesis and collagen fiber density can help rejuvenate the skin, restoring its youthful appearance and vitality [63].

Our investigation found that PLLA treatment was associated with increased TGF-β levels in aged skin, alongside heightened phosphorylation of AKT and ERK, increased expression of CDK4 and Cyclin D, and enhanced collagen synthesis. Notably, there was also an increase in PCNA-positive cells in aged skin following PLLA treatment. PLLA treatment resulted in increased levels of mTOR/S6K1/4EBP1 and Collagen I/III in aged skin. PLLA treatment also enhanced collagen fiber density and the density of newly synthesized and mature collagen fibers in aged skin.

Expanding upon prior findings that PLLA enhances collagen synthesis [2,64], our study elucidates a previously unrecognized role of Piezo1 in mediating this process. Specifically, we demonstrated that PLLA activation of Piezo1 induces fibroblast proliferation and the secretion of TGF-β from senescent fibroblasts. This highlights the intricate relationship between Piezo1, collagen synthesis, and the skin aging process.

Various mechanical stimuli, including ECM stiffness and mechanical stretching, activate Piezo1 [14,17]. While our study did not directly quantify the stiffness of PLLA, precluding a definitive comparison with native ECM stiffness, we did observe a significant increase in [Ca^2+^]_i_ levels following PLLA treatment, which was subsequently attenuated by administration of GsMTx4. Although the specific mechanical cues responsible for Piezo1 activation upon fibroblast interaction with PLLA remain uncharacterized, it is reasonable to hypothesize that the disparity in stiffness between PLLA and native ECM may contribute to Piezo1 activation.

There are several limitations to our study. First, we used GsMTx4 to inhibit Piezo1 activity. GsMTx4 could inhibit cation-permeable mechanosensitive channels such as the Piezo and Transient receptor potential (TRP) channel families [65,66,67]. TRP channels are also Ca^2+^ permeable channels [68]. Since GsMTx4 could inhibit TRP channels, we cannot completely rule out the possibility that PLLA also affected the TRP channel, resulting in changes in intracellular [Ca^2+^]_i_. Secondly, we evaluated [Ca^2+^]_i_ to investigate whether PLLA activated Piezo1. Since various factors involve in changes in [Ca^2+^]_i_ other than Piezo1, further study using a Piezo1 knock-out animal model is necessary for revealing exact effect of PLLA on activating Piezo1.

Understanding the role of ECM stiffness in skin aging and rejuvenation is crucial. Changes in ECM stiffness are known to occur during aging, contributing to the loss of skin elasticity and the formation of wrinkles [69,70,71]. Therefore, investigating how biomaterials like PLLA, with varying stiffness levels, affect Piezo1 activation and subsequent fibroblast responses could provide valuable insights into the mechanobiology underlying skin aging and rejuvenation.

Our findings suggest potential therapeutic approaches to enhance skin rejuvenation by targeting Piezo1-mediated pathways. Understanding the intricate relationship between PLLA stiffness, Piezo1 activation, and fibroblast responses offers novel strategies for guiding tissue remodeling toward rejuvenation. These insights highlight the necessity for continued research in this area, with significant potential for clinical translation. Ultimately, these advancements may revolutionize skin rejuvenation methods and improve treatment strategies for fibrotic disorders, advancing regenerative medicine practices.

## 4. Materials and Methods

### 4.1. Poly L-lactic Acid Preparation

PLLA was prepared by combining 18 g (0.12 mol) of L-lactide (Corbion, Amsterdam, The Netherlands), 0.09 mL of stannous octoate (Sigma-Aldrich, St. Louis, MO, USA), and 0.54 g (0.003 mol) of the initiator 1-dodecanol (Sigma-Aldrich) in a 1 L double-jacketed reactor at room temperature. Oxygen and moisture were removed, and nitrogen was injected for 5 min. The mixture was heated to 120 °C over 1 h and stirred for 4 h at 120 °C. Synthesized PLLA was obtained via vacuum filtration and purified with ethanol to remove unreacted substances.

PLLA was dissolved in 220 mL of dichloromethane (99.5%; Samchun Chemical Co., Ltd., Seoul, Republic of Korea) at room temperature. The solution was diluted by adding 320 mL of 1% polyvinyl alcohol (87–90%, molecular weight: 30,000–70,000; Sigma-Aldrich) dropwise while stirring at 2000 rpm with a shear mixer. After stirring, the solution was filtered under vacuum for 3 h at room temperature and centrifuged at 3000 rpm for 10 min at room temperature to pellet PLLA. After removing the supernatant, any polyvinyl alcohol that remained in the PLLA was redispersed in 50 mL distilled water (DW) and centrifuged at 3000 rpm for 10 min at room temperature. PLLA polymers were dried in an oven at 45 °C for 24 h [72,73,74,75].

### 4.2. In Vitro Experiments

#### 4.2.1. Cell Culture

CCD-986Sk fibroblasts obtained from the American Type Culture Collection (ATCC; Manassas, VA, USA) were cultured in Iscove’s Modified Dulbecco’s Medium (IMDM; Welgene, Gyeongsan, Republic of Korea) supplemented with 10% fetal bovine serum (FBS; Gibco-Thermo Fisher Scientific, Rockford, IL, USA) and 1% penicillin/streptomycin (Welgene). Cultures were maintained at 37 °C in a humidified atmosphere with 5% CO_2_.

#### 4.2.2. Experimental Design

CCD-986Sk fibroblasts were incubated with 350 μM H_2_O_2_ for 1.5 h to induce senescence. Subsequently, cells were washed with Dulbecco’s PBS (DPBS; Gibco-Thermo Fisher Scientific) and further cultured in growth medium for 72 h to induce senescent cells [76]. The senescent cells were treated with PBS, 200 μg/mL PLLA [2], 2.5 μM GsMTx4 [77], or a combination of 200 μg/mL PLLA and 2.5 μM GsMTx4, followed by an additional 24 h culture period (Figure 1A). Cell lysates were then collected using a cell scraper for further analysis.

### 4.3. In Vivo Experiments

#### 4.3.1. Mouse Model and Maintenance

C57BL/6N mice (6 weeks old) were obtained from the Central Laboratory Animal Center (Incheon, Republic of Korea) and acclimatized in our facility for 1 week before commencing experiments. Following acclimatization, mice were bred, and the offspring were raised until they reached 13 months of age. All mice were housed under controlled conditions of constant temperature (20–24 °C) and humidity (45–55%) with ad libitum access to food and water. Ethical approval for this study was obtained from the Gachon University Animal Experiment Ethics Committee (IACUC, approval number LCDI-2021-0172).

#### 4.3.2. Experimental Design

Acclimatized mice were randomly divided into two groups. Each group received injections of either 100 μL saline or PLLA (10 mg/mL) into the dermis at five sites on the backs of mice, resulting in a total injection volume of 500 μL per mouse [2]. After 28 days, back skin samples were harvested (Figure 4A).

### 4.4. Sample Preparation

#### 4.4.1. Protein Isolation

Protein isolation was conducted following the instructions provided with the EzRIPA Lysis kit (ATTO Corporation, Tokyo, Japan).

For fibroblasts, CCD-986Sk cells were washed with cold PBS and incubated with radioimmunoprecipitation assay (RIPA) lysis buffer supplemented with 1% protease inhibitor and 1% phosphatase inhibitor (ATTO Corporation) on ice for 15 min. After incubation, cells were scraped, transferred to new tubes, and centrifuged at 14,000× *g* for 15 min at 4 °C. The resulting supernatant, containing isolated protein, was transferred to new tubes.

For skin samples, 40 mg of tissue was cut into pieces and diluted with 1 mL of RIPA buffer being supplemented with 10 μL each of protease and phosphatase inhibitors. Tissues were homogenized using a Bead Homogenizer (BIOPREP-24R; Allsheng, Hangzhou, China) with 10 cycles of 40 s/60 s working/resting time and then incubated on ice for 10 min. Following 15 min of sonication (high power, 10 s/60 s working/resting time), samples were centrifuged at 14,000× *g* for 15 min at 4 °C, and supernatants were collected.

Isolated protein concentrations were determined using a bicinchoninic acid assay kit (BCA kit; Thermo Fisher Scientific, Rockford, IL, USA).

#### 4.4.2. Tissue Processing, Paraffin Embedding and Sectioning

Skin tissues were fixed in cold 4% paraformaldehyde (Sigma-Aldrich) for 72 h, followed by placement in cassettes and washing with DW. Tissues underwent processing in a tissue processor (Leica, Wetzlar, Germany), where they were sequentially immersed in 95% and 99% ethanol (Duksan, Siheung, Republic of Korea) for dehydration. Dehydrated tissues were then immersed in xylene (Duksan) for clearing and infiltrated with paraffin (Leica). Tissue blocks soaked in paraffin were then made into paraffin blocks using an embedding machine (Leica). Blocks were sectioned into 7 μm thick sections using a microtome (Leica), placed on coated slides, and incubated overnight at 60 °C to facilitate attachment to the slides.

### 4.5. Measurement of Intracellular Ca^2+^ Concentration

For the assessment of [Ca^2+^]_i_ levels, cells were transferred onto coverslips and incubated with 4 μM Fura-2-AM and 0.05% pluronic acid in physiological salt solution (PSS) for 15 min at room temperature in the dark. PSS contained 140 mM NaCl, 10 mM glucose, 1 mM MgCl_2_, 5 mM KCl, 10 mM HEPES, and 1 mM CaCl_2_ with a pH of 7.4 and an osmolality of 300 mosmo. Cells were then washed with PSS for 5 min.

[Ca^2+^]_i_ levels were determined by measuring Fura-2 fluorescence using dual excitation wavelengths of 340 nm and 380 nm, with an emission wavelength of 530 nm. [Ca^2+^]_i_ is presented as the Fura-2 fluorescence ratio (340/380). The emitted fluorescence was monitored using a CCD camera (Retiga 6000, Q-imaging, Tucson, AZ, USA) attached to an inverted microscope (Olympus), and analyzed using a Meta Fluor system (Molecular Devices, San Jose, CA, USA). The length of scale bar was measured by Meta Morph system (Molecular Devices).

### 4.6. Western Blot Analysis

For Western blot analysis, 30 μg of cell lysate or skin proteins were mixed with 4× LDS sample buffer and 10× sample-reducing agent (Thermo Fisher Scientific) and heated at 70 °C for 10 min. Denatured samples were separated by 10% sodium dodecyl sulfate-polyacrylamide gel electrophoresis (SDS-PAGE) at 200 V for 25 min using MOPS SDS running buffer (Invitrogen, Waltham, MA, USA) and transferred to polyvinylidene difluoride (PVDF) membranes (Millipore, Burlington, MA, USA) using a semi-dry transfer system (ATTO) at a current of 1 A for 10–15 min. PVDF membranes were blocked with 5% skim milk (LPS Solution, Daejeon, Republic of Korea) in 0.1% Tween 20 (SPL, Pocheon, Republic of Korea) in tris-buffered saline (TTBS) at room temperature for 1–2 h. Blocked membranes were washed three times with 0.1% TTBS and incubated with primary antibodies overnight at 4 °C (Table S1). After three washes with 0.1% TTBS, membranes were incubated with horseradish peroxidase-conjugated secondary antibodies (Vector Laboratories, Newark, CA, USA) for 1 h at room temperature.

Protein bands were visualized using ECL Select^TM^ Western Blotting Detection Reagent (Cytiva, Incheon, Republic of Korea) and imaged using a ChemiDoc Imaging System (Bio-Rad, Hercules, CA, USA). For quantitative protein analysis, band intensities were quantified using ImageJ software version 1.53s (NIH). Equal loading was assessed by the intensity of β-actin bands. Each group was compared to a reference sample [78].

### 4.7. Enzyme-Linked Immunosorbent Assays

To coat wells, 96-well microplates were incubated overnight at 4 °C with 0.05 M carbonate–bicarbonate buffer (pH 9.6). Microplates were washed three times with 0.1% Tween 20 in PBS (TPBS). To prevent non-specific binding, microplates were incubated with 5% skim milk (LPS Solution) in 0.1% TPBS overnight at 4 °C and subsequently washed three times with 0.1% TPBS.

Cell lysate, supernatant, or skin protein samples (30 μg) were added to each well and incubated overnight at 4 °C. Wells were then washed with 0.1% TPBS and incubated overnight at 4 °C with primary antibodies diluted in PBS (Table S1). After subsequent washing with 0.1% TPBS, horseradish peroxidase-conjugated secondary antibodies (Vector Laboratories) were added and incubated at room temperature for 4 h.

For visualization, tetramethylbenzidine solution (Sigma-Aldrich) was applied to each well and incubated for 15–20 min at room temperature. Reactions were stopped with 1 N sulfuric acid (Sigma-Aldrich). Absorbance measurements were taken at 450 nm using a microplate reader (Thermo Fisher Scientific).

### 4.8. Staining

#### 4.8.1. Immunohistochemistry

Slides were deparaffinized and rehydrated through sequential transfers to xylene and 100–70% ethanol. Following three washes with PBS, slides were blocked by incubation with a serum solution for 1 h at room temperature and then incubated with primary antibodies overnight at 4 °C (Table S1). After washing with PBS, slides were incubated with biotinylated secondary antibodies (Vector Laboratories) for 1 h at room temperature. Slides were rinsed with PBS, treated with an ABC reagent (Vector Laboratories) to enhance sensitivity, and incubated with a 3,3′-diaminobenzidine solution (Sigma-Aldrich) for 5 min to develop a brown reaction. Slides were then incubated with hematoxylin (KPNT, Cheongju, Republic of Korea) for 30 s, washed with DW, dehydrated, and mounted using a DPX mounting solution (Sigma-Aldrich).

#### 4.8.2. Masson Trichrome Staining

Masson trichrome stain was conducted following the instructions provided with the Trichrome Stain Kit (Modified Masson’s; Scytek Laboratories, West Logan, UT, USA). Briefly, tissue slides were deparaffinized, hydrated in DW, incubated in preheated Bouin’s Fluid (Scytek Laboratories) for 1 h at 60 °C, and washed with DW. The staining process involved sequential immersion in the following solutions: working Weigert’s Iron Hematoxylin (Scytek Laboratories) for 5 min, Biebrich Scarlet/Acid Fuchsin Solution (Scytek Laboratories) for 3 min, Phosphomolybdic/Phosphotungstic Acid Solution (Scytek Laboratories) for 12 min, Aniline Blue Solution (Scytek Laboratories) for 3 min, and Acetic Acid Solution (Scytek Laboratories) for 5 min at room temperature. Each reagent was followed by a DW wash, except for the Phosphomolybdic/Phosphotungstic Acid Solution. Following the staining procedure, slides were dehydrated and mounted using DPX mounting solution (Sigma-Aldrich).

#### 4.8.3. Herovici Staining

Herovici staining was performed following the instructions provided with the Herovici Stain Kit (Scytek Laboratories). Tissue slides were deparaffinized and hydrated with DW. Slides were then incubated in Hematoxylin Weigert’s Iron (Scytek Laboratories) for 8 min at room temperature and washed with DW. Next, slides were incubated in Herovici Solution for 2 min at room temperature, washed with DW, dehydrated, and mounted using a DPX mounting solution (Sigma-Aldrich).

#### 4.8.4. Quantitative Analysis and Procedure of Scanning and Image Selection

All stained slides were imaged using a slide scanner (Motic Scan Infinity 100; Motic, Vancouver, BC, Canada), and images were captured at a size of 1 × 1 cm^2^ area. The color representing positive staining (brown for immunohistochemistry, blue for Masson trichrome staining, and blue and red for Herovici staining [34,35]) was extracted from the images and converted to black to quantify staining intensity. Groups were compared with a saline control sample [79,80].

### 4.9. Statistical Analysis

The Kruskal–Wallis test was conducted to compare all groups, followed by the Mann–Whitney U test for post hoc comparisons. Results are presented as the mean ± standard deviation. All statistical analyses were performed using SPSS version 26 (IBM, Armonk, NY, USA).

## 5. Conclusions

In conclusion, our study elucidates the pivotal role of PLLA in modulating fibroblast behavior and driving tissue remodeling processes. Our findings highlight the intricate molecular mechanisms underlying PLLA-mediated rejuvenation of senescent fibroblasts. Specifically, we demonstrated that PLLA initiates a signaling cascade by activating Piezo1, subsequently leading to the upregulation of critical pathways including ERK1/2, AKT, and mTOR/S6K1/4EBP. These signaling events culminate in enhanced proliferation of senescent fibroblasts, suggesting a robust rejuvenation effect.

## Data Availability

All data are contained within this article.

## Supplementary Materials

The supporting information can be downloaded at: https://www.mdpi.com/article/10.3390/ijms25137232/s1.
