# Supplementary material for: Piezo1 Activation Drives Enhanced Collagen Synthesis in Aged Animal Skin Induced by Poly L-Lactic Acid Fillers
Source: Int J Mol Sci. 2024 Jun 30;25(13):7232. doi: 10.3390/ijms25137232 (PMC11242599; doi:10.3390/ijms25137232)
Supplement: Supplementary file 1 [file ijms-25-07232-s001.zip › ijms-3040743-supplementary.pdf]

International Journal of *Molecular Sciences*

MDPI

Article

Piezo1 Activation Drives Enhanced Collagen Synthesis in Aged Animal Skin Induced by Poly L-Lactic Acid Fillers

Kyung-A Byun [1,2,3,†], Je Hyuk Lee [1,4,†], So Young Lee [5], Seyeon Oh [3], Sosorburam Batsukh [1,3], Gwahn-woo Cheon [1,6], Dongun Lee [7], Jeong Hee Hong [7], Kuk Hui Son [5,*] and Kyunghee Byun [1,3,7,*]

[1] Department of Anatomy & Cell Biology, College of Medicine, Gachon University, Incheon 21936, Republic of Korea
[2] LIBON Inc., Incheon 22006, Republic of Korea
[3] Functional Cellular Networks Laboratory, Lee Gil Ya Cancer and Diabetes Institute, Gachon University, Incheon 21999, Republic of Korea
[4] Doctorbom Clinic, Seoul, 06614, Republic of Korea
[5] Department of Thoracic and Cardiovascular Surgery, Gachon University Gil Medical Center, Gachon University, Incheon 21565, Republic of Korea
[6] Maylin Clinic, Pangyo 13529, Republic of Korea
[7] Department of Health Sciences and Technology, Gachon Advanced Institute for Health & Sciences and Technology (GAIHST), Gachon University, Incheon 21999, Republic of Korea; minicleo@gachon.ac.kr (J.H.H.)
* Correspondence: dr632@gachon.ac.kr (K.H.S.); khbyun1@gachon.ac.kr (K.B.); Tel.: +82-32-460-3666 (K.H.S.); +82-32-899-6511 (K.B.)
† These authors contributed equally to this work.

Citation: Byun, K.-A.; Lee, J.H.; Lee, S.Y.; Oh, S.; Batsukh, S.; Cheon, G.-w.; Lee, D.; Hong, J.H.; Son, K.H.; Byun, K. Piezo1 Activation Drives Enhanced Collagen Synthesis in Aged Animal Skin Induced by Poly L-Lactic Acid Fillers. *Int. J. Mol. Sci.* **2024**, *25*, 7232. https://doi.org/10.3390/ijms25137232

Academic Editor: Andrzej Slominski

Received: 18 May 2024
Revised: 28 June 2024
Accepted: 28 June 2024
Published: 30 June 2024

Copyright: © 2024 by the authors. Licensee MDPI, Basel, Switzerland. This article is an open access article distributed under the terms and conditions of the Creative Commons Attribution (CC BY) license (https://creativecommons.org/licenses/by/4.0/).

Table S1. List of antibodies for Western blot (WB), ELISA, and IHC.

Antibody	Company	Catalog No.	Dilution rate		
			WB	ELISA	IHC
β-actin	Cell Signaling Technology	4967	1:1,000		
Piezo1	proteintech	15939-1-AP	1:500		
pERK1/2 (Thr202/Tyr204)	Cell Signaling Technology	9101s	1:1,000		
ERK1/2	Cell Signaling Technology	9102s	1:1,000		
pAKT (Ser473)	proteintech	66444-1-lg	1:1,000		
AKT	BD Biosciences	610860	1:1,000		
CDK4	proteintech	11026-1-ap	1:1,000		
Cyclin D1	BioLegend	681902	1:1,000		
pmTOR (Ser2448)	Abbexa	Abx329502	1:1,000		
mTOR	Sigma-Aldrich	T2949	1:1,000		
pS6K1 (Thr389)	Cell Signaling Technology	9205s	1:1,000		
S6K1	Cell Signaling Technology	34475s	1:1,000		
p4EBP1 (Thr37/46)	Cell Signaling Technology	2855s	1:1,000		
4EBP1	Cell Signaling Technology	9644s	1:1,000		
TGF-β	Abcam	Ab64715		1:500	
Collagen I	Santa Cruz Biotechnology	sc-293182		1:1,000	1:50
Collagen III	Bioss Antibodies	bs-0549R		1:1,000	1:100
PCNA	Abcam	ab18197			1:500

Disclaimer/Publisher's Note: The statements, opinions and data contained in all publications are solely those of the individual author(s) and contributor(s) and not of MDPI and/or the editor(s). MDPI and/or the editor(s) disclaim responsibility for any injury to people or property resulting from any ideas, methods, instructions or products referred to in the content.
